# Sacituzumab tirumotecan in an advanced HR+/HER2-breast cancer patient with bone marrow metastasis-induced refractory thrombocytopenia: a case report and literature review

**DOI:** 10.3389/fphar.2026.1854219

**Published:** 2026-07-06

**Authors:** Jing Han, Juwei Gao, Fei Dou, Yinyin Ying, Zhaoyi Xu, Hairu Mao, Weiping Zhang, Qinglin Zhang

**Affiliations:** 1 The Third Affiliated Hospital of Zhejiang Chinese Medical University, Hangzhou, China; 2 Zhejiang Chinese Medical University, Hangzhou, China

**Keywords:** antibody–drug conjugate, bone marrow metastases, breast cancer, sac-TMT, thrombocytopenia, trop-2

## Abstract

**Background:**

Bone marrow metastasis (BMM) is an adverse prognostic factor in advanced breast cancer and may cause severe myelosuppression, particularly refractory thrombocytopenia, thereby limiting systemic therapy. Sacituzumab tirumotecan (sac-TMT) is a Trop-2-directed antibody-drug conjugate (ADC), but evidence for its use in patients with severe thrombocytopenia and compromised bone marrow reserve remains limited. We report a real-world case of sac-TMT use in this challenging clinical setting and discuss relevant considerations regarding ADC resistance and treatment sequencing.

**Case Presentation:**

A 62-year-old postmenopausal woman (BMI: 16.23 kg/m^2^), with hypertension, hyperlipidemia, and no known family history of hereditary disease, was diagnosed in March 2015 with HR+/HER2 − breast cancer (pT1N2M0). She underwent right mastectomy with axillary dissection and immediate latissimus dorsi flap reconstruction, followed by four cycles of TC chemotherapy, adjuvant radiotherapy, and 6 years of tamoxifen. She relapsed in July 2021 with multiple bone metastases; after multiple lines of endocrine therapy and CDK4/6 inhibitors, her disease progressed. In September 2024 she was diagnosed with bone marrow metastases accompanied by severe refractory thrombocytopenia (nadir 12 × 10^9/L), unresponsive to conventional platelet-promoting therapies. In December 2024, sac-TMT was initiated together with platelet-supportive measures; during therapy platelets stabilized at ≥50 × 10^9/L, with no grade ≥3 non-hematologic adverse events, and disease stability exceeded 8 months.

**Conclusion:**

Sac-TMT combined with intensive hematologic supportive care was feasible in this heavily pretreated HR+/HER2 − breast cancer patient with BMM-induced refractory thrombocytopenia. Platelet stabilization, reduced transfusion dependence, and clinical disease stability were observed before PET-CT-confirmed progression. Further case series and prospective studies are needed to validate the feasibility and safety of this approach in similar high-risk patients.

## Background

Breast cancer is a malignant tumor arising in breast tissue, primarily due to abnormal proliferation of mammary epithelial cells; it is the most common malignancy among women globally and a leading cause of cancer-related death (Montazeri Aliabadi, 2024). Hormone receptor–positive (HR+)/(HER2-) cases account for approximately 70% of breast cancer diagnoses and represent the most common subtype (Sledge et al., 2017). This subtype is relatively indolent biologically; in early disease, surgery plus endocrine therapy can yield favorable outcomes, yet about 30% of early breast cancer patients progress to advanced disease, and the survival rate for advanced breast cancer is only 20% (Wang et al., 2024). The skeleton is the most common site of distant metastasis for this subtype; studies show HR+/HER2-tumors tend to develop bone-only metastases, whereas HER2-positive and triple-negative subtypes are more likely to involve visceral and brain metastases (Zhang et al., 2025). BMM disrupts the hematopoietic microenvironment, leading to anemia, leukopenia, and thrombocytopenia, severely limiting subsequent treatments and indicating a very poor prognosis. For advanced HR+/HER2-breast cancer, international and domestic guidelines recommend endocrine therapy plus CDK4/6 inhibitors as first-line treatment (Huang et al., 2022). However, once resistance develops, subsequent options are limited and efficacy declines line by line. In patients with severely compromised marrow reserve, the broad cytotoxicity of conventional chemotherapy exacerbates myelosuppression and is often abandoned (Li et al., 2024; Hussain et al., 2025). Collectively, these challenges highlight an urgent need for effective therapies that maintain antitumor activity while minimizing hematologic toxicity, particularly in patients with compromised bone marrow function.

Antibody–drug conjugates (ADCs), a major advance in precision oncology, deliver potent cytotoxic agents *via* monoclonal antibodies, thereby enhancing targeting and efficacy (Wang et al., 2025). Several ADCs have achieved meaningful progress in breast cancer, including HER2-directed ADCs such as trastuzumab emtansine (T-DM1) and trastuzumab deruxtecan (T-DXd), as well as Trop-2-directed ADCs such as sacituzumab govitecan (SG) and sacituzumab tirumotecan (sac-TMT) (Huang et al., 2024; Bardia et al., 2024). SG is a Trop-2-directed ADC linked to SN-38, the active metabolite of irinotecan (Kang, 2024). By contrast, sac-TMT (MK-2870/SKB264) is a distinct Trop-2-directed ADC composed of a humanized anti-Trop-2 monoclonal antibody, a sulfonyl-pyrimidine–based linker, and a belotecan-derived topoisomerase I inhibitor payload, KL610023 (Sofianidi et al., 2026; Ouyang et al., 2025). Therefore, although SG and sac-TMT both target Trop-2, their linker-payload structures and clinical evidence should be considered separately. TROP-2 is highly expressed in a substantial proportion of HR+/HER2-breast cancers and has been associated with aggressive tumor behavior, making Trop-2-directed ADCs a biologically relevant therapeutic strategy in this population (Rugo et al., 2020).

The strongest phase III evidence for Trop-2-directed ADC therapy in HR+/HER2− metastatic breast cancer currently comes from SG rather than sac-TMT. In the TROPiCS-02 trial, SG significantly improved progression-free survival and overall survival compared with physician’s-choice chemotherapy in heavily pretreated HR+/HER2− metastatic breast cancer (Rugo et al., 2020; Rugo et al., 2023). Its safety profile was generally manageable, although hematologic adverse events, particularly neutropenia and anemia, were common (Rugo et al., 2023; Ouyang et al., 2025; Garrigos et al., 2024). Importantly, these efficacy and safety data are specific to SG and should not be directly extrapolated to sac-TMT.

Sac-TMT-specific clinical evidence remains more limited. In the phase 1/2 MK-2870–001/KL264-01 study, sac-TMT showed encouraging antitumor activity in previously treated breast cancer, including an objective response rate of 31.7% in the HR+/HER2− cohort, with a manageable safety profile (Ouyang et al., 2025). More recently, the randomized phase 3 OptiTROP-Breast01 trial demonstrated that sac-TMT improved progression-free survival compared with chemotherapy in previously treated metastatic triple-negative breast cancer; however, these data were generated in TNBC and should not be directly extrapolated to HR+/HER2− disease with BMM and severe cytopenias (Yin et al., 2025). Nevertheless, patients with severe baseline cytopenias or active bone marrow failure are generally underrepresented or excluded from prospective trials. Therefore, the feasibility of sac-TMT in patients with bone marrow metastasis-induced refractory thrombocytopenia remains uncertain and is the central clinical question illustrated by the present case.

## Case presentation

The patient was a 62-year-old postmenopausal woman (BMI: 16.23 kg/m^2^) with a medical history of hypertension for over 1 year and hyperlipidemia for more than 2 years, and no known family history of hereditary diseases. In March 2015, she underwent right mastectomy with axillary lymph node dissection and immediate latissimus dorsi flap reconstruction for a right breast mass. Postoperative pathology revealed invasive ductal carcinoma measuring 1.4 × 0.7 cm, grade I, with 4 of 14 axillary lymph nodes positive. Immunohistochemistry showed ER 90% positive, PR 5% positive, HER2 0, and Ki-67 5%, consistent with a Luminal A–like subtype, stage pT1N2M0. The patient subsequently received four cycles of adjuvant TC chemotherapy (docetaxel 75 mg/m^2^ IV on day 1 plus cyclophosphamide 600 mg/m^2^ IV on day 1, every 3 weeks), followed by chest wall and supraclavicular radiotherapy, and endocrine therapy with tamoxifen (20 mg orally once daily) for 6 years.

Overall, the patient initially achieved standard-of-care management with favorable disease control in the early stage.

In July 2021, the patient developed rib pain, and PET-CT revealed a subcutaneous nodule at the surgical site along with multiple bone metastases involving the ribs, vertebrae, and pelvis. First-line endocrine therapy with fulvestrant (500 mg intramuscularly on days 1 and 15, followed by every 4 weeks) was initiated, achieving disease stability for 5 months before discontinuation due to cerebral infarction. In April 2022, treatment was switched to palbociclib combined with letrozole and zoledronic acid. In September 2022, the patient underwent vertebroplasty for an L2 vertebral fracture, with pathology confirming metastatic disease (ER 90%, PR 90%, HER2 0, Ki-67 10%, BRCA negative). Due to disease progression, fulvestrant plus abemaciclib (100 mg) was initiated in November 2022. In September 2023, owing to treatment intolerance, the regimen was modified to fulvestrant plus dalpiciclib (100 mg), and bone-modifying therapy was switched to denosumab.

These findings indicate a typical pattern of sequential endocrine therapy and CDK4/6 inhibitor–based treatment, with gradually diminishing efficacy over time.

In July 2024, disease progression was observed on bone scintigraphy. On 26 September 2024, PET-CT demonstrated a recurrent lesion at the surgical site and diffuse abnormal bone marrow metabolism with osteoblastic changes, consistent with bone marrow metastasis (BMM). During dalpiciclib treatment, the patient developed severe thrombocytopenia, with a nadir platelet count of 12 × 10^9^/L, which did not improve despite dose reduction to 50 mg. Although capecitabine chemotherapy was considered, it could not be initiated due to persistent cytopenias.

At this stage, disease progression was accompanied by significant bone marrow involvement and severe hematologic compromise, markedly limiting available therapeutic options.

From October 2024 onward, the patient required multiple hospitalizations and received intensive hematologic supportive treatments, including romiplostim (1 μg/kg subcutaneously weekly), eltrombopag (25 mg orally once daily), avatrombopag (20 mg orally once daily), recombinant human thrombopoietin, and platelet transfusions (a total of 16 units), all with limited efficacy. Bone marrow biopsy ultimately confirmed metastatic poorly differentiated carcinoma, establishing that the refractory thrombocytopenia was secondary to extensive marrow infiltration.

These findings highlight the clinical challenge of managing advanced breast cancer complicated by bone marrow metastasis–induced refractory thrombocytopenia, necessitating alternative therapeutic strategies with improved hematologic safety.

## Pathological findings

## Pathological diagnosis

**FIGURE 1:**
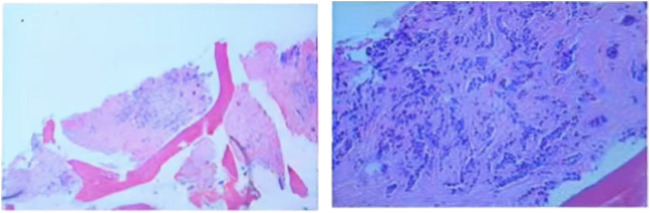
Histopathological and staining findings from the bone marrow core biopsy. HE and PAS staining showed infiltrative atypical tumor cell proliferation within the bone marrow, and reticulin staining showed grade 3 fibrosis. The findings supported metastatic poorly differentiated carcinoma involving the bone marrow.

(Marrow biopsy tissue) Metastatic poorly differentiated carcinoma (Figure 1).

HE and PAS staining show atypical cell proliferation within the bone marrow tissue, exhibiting infiltrative growth in a cord-like pattern. Reticulin fiber staining (MF-3 grade).

Special Staining: Specimen 202463121-1: PAS special staining (+), Reticulin staining (grade 3).

**FIGURE 2:**
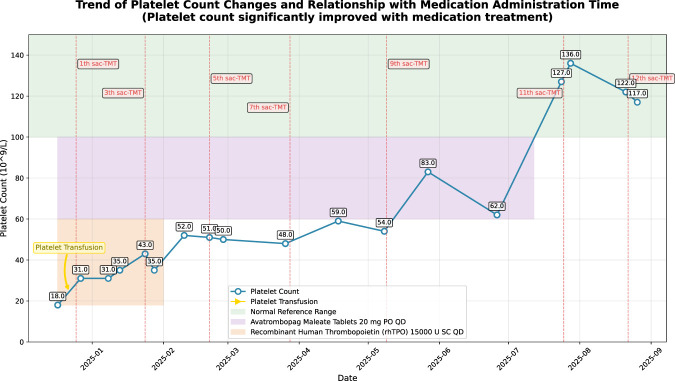
Longitudinal platelet count changes during sacituzumab tirumotecan treatment and concurrent hematologic supportive care. The blue line indicates platelet counts, red dashed lines indicate sac-TMT administrations, and shaded areas indicate periods of thrombopoietic supportive therapy. Platelet stabilization occurred in the setting of combined antitumor treatment and intensive hematologic support.

**FIGURE 3:**
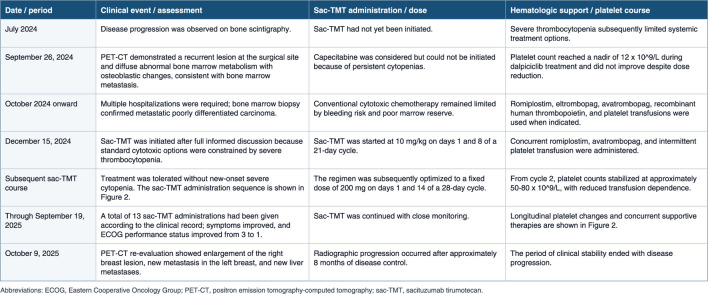
Clinical timeline of sac-TMT treatment, supportive care, and disease assessment.

Given late-line breast cancer with severe thrombocytopenia and bleeding risk, conventional chemotherapy was limited; the patient and family were motivated for treatment. After full informed discussion, sac-TMT was initiated on 15 December 2024. Considering the patient’s poor bone marrow reserve and low body weight (BMI: 16.23 kg/m^2^), we commenced with a reduced and modified regimen of sac-TMT (sacituzumab tirumotecan) at 100 mg on days 1 and 8 of a 21-day cycle, alongside romiplostim, avatrombopag, and intermittent platelet transfusion. Following the first cycle, the patient tolerated the treatment well without new-onset severe cytopenia. To improve convenience and adherence, the regimen was subsequently optimized to a fixed dose of 200 mg administered on days 1 and 14 of a 28-day cycle, which was then maintained. A 5-HT3 receptor antagonist and dexamethasone were used before the first infusion to prevent reactions; each infusion lasted about 60 min with good monitoring. Tolerability was good, with no grade ≥3 non-hematologic adverse reactions. From cycle 2, platelets stabilized at 50–80 × 10^9/L, with marked reduction in transfusion dependence (Table 1; Figure 2). The key clinical timeline, including sac-TMT initiation, subsequent dose-schedule adjustment, hematologic supportive interventions, imaging assessments, best clinical assessment, and progression, is summarized in Figure 3.

**TABLE 1:** Safety indicators-laboratory values.

Laboratory values	WBC (*10^9/L)	NEU% (*10^9/L)	Hb(g/L)	ALT(U/L)	AST(U/L)	Urea(U/L)	Cr(μmol/L)
2024.12.17	3.91	56.8	72	92.7	57.4	3.29	70.5
2025.03.26	3.67	60	67	51.4	71.6	7.29	55.2
2025.09.23	3.06	70.2	103	22.2	82.9	4.67	53.4

**FIGURE 4:**
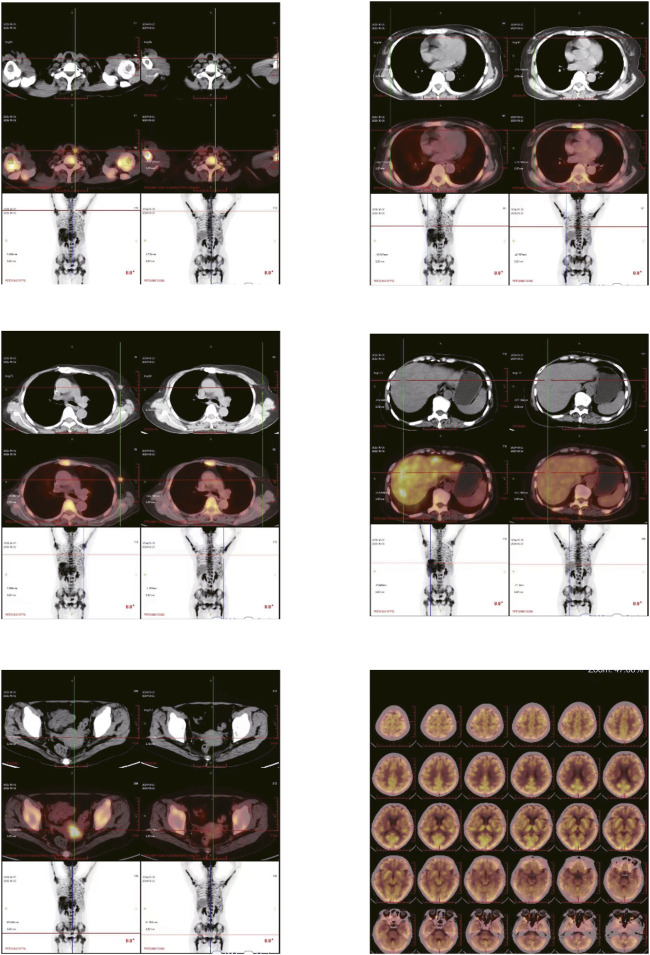
Comparison of pre- and post-treatment PET-CT findings. Baseline PET-CT showed diffuse abnormal bone marrow metabolism with osteoblastic changes consistent with bone marrow metastasis. PET-CT re-evaluation on 9 October 2025 showed enlargement of the right breast lesion, a new left breast lesion, and new liver metastases, indicating disease progression.

By 19 September 2025, a total of 13 sac-TMT administrations had been given according to the clinical record; symptoms improved, and ECOG performance status improved from 3 to 1. Response was not assessed according to formal RECIST criteria; instead, disease control was evaluated using a composite clinical assessment based on symptom improvement, ECOG performance status, platelet stabilization and reduced transfusion dependence, together with available PET-CT-based radiographic information. The best clinical assessment during this period was disease stability. On 9 October 2025, PET-CT re-evaluation showed enlargement of the right breast lesion, new metastasis in the left breast, and new liver metastases, indicating disease progression (Figure 4). Thus, disease control was maintained for approximately 8 months before radiographic progression.

## Discussion and literature review

Bone marrow metastasis (BMM) from breast cancer is relatively uncommon in routine clinical practice and is usually observed in the advanced-stage diseases (Zhang et al., 2023). Its clinical presentation is often characterized by anemia, thrombocytopenia, and bone pain, whereas leukocyte counts may remain relatively preserved. In patients with advanced HR+/HER2− breast cancer, progressive cytopenias—particularly severe thrombocytopenia developing during the course of metastatic progression—should raise strong suspicion for bone marrow involvement rather than being attributed solely to treatment-related myelosuppression (Yang et al., 2022; Xiao et al., 2009). In the present case, the patient had a long disease course after initial standard treatment and subsequently developed diffuse skeletal metastases, followed by persistent and profound thrombocytopenia during later-line therapy. PET-CT demonstrated diffuse abnormal bone marrow metabolism with osteoblastic changes, and bone marrow biopsy ultimately confirmed metastatic poorly differentiated carcinoma. These findings underscore the importance of early recognition of BMM in patients with unexplained or disproportionate hematologic deterioration, because delayed diagnosis may further narrow already limited treatment opportunities.

This case also highlights the major therapeutic challenge posed by BMM-associated refractory thrombocytopenia. The patient had previously received multiple lines of endocrine-based therapy and CDK4/6 inhibitor-containing regimens, which represent the standard sequential treatment strategy for HR+/HER2− metastatic breast cancer (Yang et al., 2022; Xiao et al., 2009). However, as the disease progressed, the duration of treatment benefit gradually shortened, and the development of extensive marrow infiltration markedly altered the subsequent therapeutic landscape. During dalpiciclib treatment, the patient’s platelet count dropped to a nadir of 12 × 10^9^/L and remained critically low despite dose reduction. For cancer-related thrombocytopenia, after exclusion of immune-related, drug-induced, and infectious causes, individualized supportive strategies may be considered, including transfusion support and thrombopoietin receptor agonists (TPO-RAs) such as romiplostim, eltrombopag, and avatrombopag (Soff et al., 2024). Although our patient received intensive hematologic supportive therapy, including TPO-RAs, recombinant human thrombopoietin, and repeated platelet transfusions, thrombocytopenia improved only minimally. More importantly, bone marrow biopsy clearly demonstrated that the cytopenia was primarily caused by extensive replacement of marrow space by tumor cells rather than reversible drug-related myelosuppression alone. In this setting, supportive therapy alone is often insufficient, and hematologic recovery largely depends on effective tumor control.

A key clinical implication of this case is that once bone marrow reserve is severely compromised, many conventional systemic therapies become difficult to administer (Rahmat and Ikhwan, 2018). Although capecitabine was considered after disease progression, it could not be initiated because of persistent thrombocytopenia. Thus, the patient entered a particularly challenging stage: on the one hand, progressive disease required further antitumor treatment; on the other hand, available therapeutic options were markedly limited by poor hematologic tolerance. This reflects a common clinical dilemma in patients with BMM: although standard chemotherapy may provide antitumor activity, it is often difficult to administer safely, whereas endocrine therapy or targeted therapy may show diminishing efficacy after multiple prior lines. Therefore, in this clinical context, alternative treatment strategies that can balance tumor control with hematologic safety are urgently needed.

Against this background, ADCs may offer a promising therapeutic option (Alevizopoulos et al., 2025). In the present case, sac-TMT was selected based on its potential to maintain antitumor efficacy while avoiding the pronounced myelosuppression commonly associated with conventional chemotherapy. Notably, after initiation of sac-TMT, the patient’s platelet count stabilized at approximately 50–80 × 10^9^/L, transfusion dependence was reduced, and treatment continuity was maintained. Although hematologic recovery was incomplete, this change was clinically meaningful, as it suggests that tumor control may have alleviated marrow infiltration to some extent and contributed to partial restoration of hematopoietic function. However, because the patient concurrently received intensive hematologic supportive care, including romiplostim, avatrombopag, recombinant human thrombopoietin, and platelet transfusions, platelet stabilization should be interpreted as the result of combined disease control and supportive measures rather than the effect of sac-TMT alone. From this perspective, our case provides real-world evidence supporting the potential feasibility of ADC therapy in heavily pretreated patients with advanced HR+/HER2− metastatic breast cancer complicated by BMM and refractory thrombocytopenia who are unable to tolerate conventional chemotherapy.

The clinical course of this patient should be interpreted in the context of the broader but still evolving evidence for Trop-2-directed ADCs. The most robust randomized evidence in HR+/HER2− metastatic breast cancer is derived from SG in the TROPiCS-02 trial, whereas sac-TMT is a distinct Trop-2 ADC with separate early-phase clinical data (Rugo et al., 2020; Rugo et al., 2023; Ouyang et al., 2025). The phase one/2 MK-2870–001/KL264-01 study reported promising activity of sac-TMT in previously treated HR+/HER2− breast cancer, but those data do not specifically address patients with overt bone marrow metastasis and severe refractory thrombocytopenia (Ouyang et al., 2025). Accordingly, our case should be viewed as a single-patient feasibility observation rather than direct confirmation of trial-level efficacy or safety in this high-risk population.

For patients such as ours, whose treatment options are severely restricted by bone marrow dysfunction, Trop-2-directed ADCs may represent a clinically relevant option, although evidence in this setting remains limited. In the present case, platelet stabilization and reduced transfusion dependence were observed after sac-TMT initiation together with intensive hematologic supportive care, suggesting that the favorable hematologic course may have reflected combined disease control and supportive measures rather than the antitumor agent alone.

Nevertheless, acquired resistance to ADC therapy remains an important limitation and should be discussed as a broader consideration prompted by this case, rather than as a mechanism directly demonstrated in this patient. In the present case, disease progression eventually occurred after an initial period of clinical disease control; however, no molecular or functional assays were performed to determine the specific mechanism of resistance. Based on previous studies and recent reviews, potential mechanisms of ADC resistance may include downregulation of target antigen expression, impaired internalization, abnormal intracellular payload release, enhanced drug efflux, altered payload sensitivity, and activation of bypass signaling pathways (Valle et al., 2025; Guidi et al., 2023). In patients with severe bone marrow dysfunction, treatment after ADC progression becomes even more challenging, as many subsequent therapeutic options may no longer be feasible. This highlights the potential value of predictive biomarkers and dynamic monitoring, such as TROP-2 expression, ctDNA analysis, and other resistance-related molecular alterations, although these strategies were not evaluated in the present case (Cursano et al., 2023; Cardillo et al., 2020; Agostinetto and Ignatiadis, 2023).

Similarly, the discussion of treatment sequencing after ADC progression should be interpreted as a forward-looking consideration rather than a conclusion derived from this single case. Next-generation ADCs, dual-target ADCs, and combination strategies involving immunotherapy, endocrine therapy, or pathway-targeted agents may provide future directions for overcoming ADC resistance (McLaughlin et al., 2025; Larose et al., 2025). For patients with HR+/HER2− or HER2-low disease, agents such as trastuzumab deruxtecan may represent potential subsequent treatment options in selected cases (Irelli et al., 2025; Modi et al., 2026). However, both the evidence for sequencing different ADCs and the safety of these strategies in patients with overt bone marrow failure and severe thrombocytopenia remain extremely limited. Therefore, the optimal sequencing strategy for this highly vulnerable population remains undefined and requires further investigation.

Several limitations of this study should also be acknowledged. First, this is a single-case observation, and its generalizability is inherently limited. Second, given the complexity of the patient’s prior treatment history and the high disease burden, it is difficult to fully disentangle the relative contributions of tumor control, supportive care, and tumor biology to the observed hematologic improvement. Third, case-specific TROP-2 expression testing was not performed, and no dynamic assessment of biomarkers related to ADC sensitivity or resistance was available during treatment. Therefore, the discussion of ADC resistance mechanisms and subsequent sequencing strategies should be interpreted as broader literature-based considerations prompted by this case, rather than findings demonstrated in this patient. Nevertheless, this case remains clinically informative, as it reflects a real-world scenario in which treatment decisions were dominated by profound bone marrow dysfunction. It should also be emphasized that this was a highly selected patient treated under intensive monitoring and multidisciplinary supportive care; therefore, the feasibility observed in this case should not be interpreted too broadly without further validation in additional case series or prospective studies. Recent literature on solid-tumor bone marrow metastasis, luminal breast cancer with disseminated carcinomatosis of the bone marrow, and Trop-2-targeted therapy further supports the need for careful patient selection, standardized hematologic-support documentation, and biomarker-aware evaluation in future studies (Yang et al., 2024; Pangarsa et al., 2024; Hu et al., 2024).

Overall, this case highlights the clinical challenge of treating advanced HR+/HER2− breast cancer with bone marrow metastasis-induced refractory thrombocytopenia. It suggests that sac-TMT, when combined with intensive hematologic supportive care, may be a feasible therapeutic option in selected patients who cannot tolerate conventional chemotherapy. However, further evidence is needed to define the safety, efficacy, and optimal sequencing of ADC therapy in this high-risk population.

## Conclusion

Sac-TMT combined with intensive hematologic supportive care was feasible in this heavily pretreated HR+/HER2− breast cancer patient with bone marrow metastasis-induced refractory thrombocytopenia. Platelet stabilization, reduced transfusion dependence, and clinical disease stability were observed before PET-CT-confirmed progression. Further case series and prospective studies are needed to validate the feasibility and safety of this approach in similar high-risk patients.

## Data Availability

The original contributions presented in the study are included in the article. Further inquiries can be directed to the corresponding authors. The raw clinical data are not publicly available due to patient privacy and confidentiality, but may be made available by the corresponding authors upon reasonable request and subject to applicable ethical and institutional restrictions.
